# Association of Metabolically Healthy Overweight Phenotype With Abnormalities of Glucose Levels and Blood Pressure Among Chinese Adults

**DOI:** 10.1001/jamanetworkopen.2019.14025

**Published:** 2019-10-25

**Authors:** Renying Xu, Xiang Gao, Yanping Wan, Zhuping Fan

**Affiliations:** 1 Ren Ji Hospital, School of Medicine, Department of Clinical Nutrition, Shanghai Jiao Tong University, Shanghai, China; 2 Department of Nutritional Sciences, The Pennsylvania State University, University Park; 3 Ren Ji Hospital, School of Medicine, Department of Digestion, Shanghai Jiao Tong University, Shanghai, China; 4 Ren Ji Hospital, School of Medicine, Department of Health Management, Shanghai Jiao Tong University, Shanghai, China

## Abstract

**Importance:**

Whether the metabolically healthy overweight (MHO) phenotype is resistant to metabolic abnormalities remains unknown.

**Objective:**

To evaluate the association of MHO with glucose level abnormalities and high blood pressure (BP) in Chinese adults.

**Design, Setting, and Participants:**

This prospective cohort study was conducted from January 1, 2013, to October 31, 2018, in the Health Management Center at Ren Ji Hospital, Shanghai, China, using data from 55 155 recruited Chinese adults. Body weight, fasting blood glucose (FBG) level, hemoglobin A_1c_ (HbA_1c_) level, and BP were measured annually.

**Exposures:**

Metabolically healthy overweight was defined as a body mass index (calculated as weight in kilograms divided by height in meters squared) of 24.0 in 2013 (baseline) and 2014, no history of metabolic diseases, and normal FBG level, HbA_1c_ level, BP, lipid profile, serum uric acid level, and liver ultrasonographic findings at baseline; the remaining participants were defined as being metabolically healthy normal.

**Main Outcomes and Measures:**

Glucose level abnormality was confirmed if the FBG level was 101 mg/dL or greater or the HbA_1c_ level was 5.7%, and high BP was confirmed if the systolic BP was 130 mm Hg or higher or the diastolic BP was 80 mm Hg or higher at least twice during the subsequent 4 years of follow-up.

**Results:**

A total of 3204 metabolically healthy Chinese adults (mean [SD] age, 39.8 [10.9] years; 1940 women [60.5%]) were included in the study. The prevalence of MHO was 7.0%. A total of 146 incident cases of glucose level abnormality and 220 cases of high BP during 4 years of follow-up were identified. Compared with the metabolically healthy normal group, the MHO group had a higher risk of glucose level abnormality (adjusted hazard ratio, 2.36; 95% CI, 1.52-3.64) and high BP (adjusted hazard ratio, 1.73; 95% CI, 1.18-2.53) after adjusting for several potential confounders.

**Conclusions and Relevance:**

Metabolically healthy overweight may be associated with a high future risk of glucose abnormality and high BP in Chinese adults. If the individuals are confirmed with MHO, early interventions, including diet and exercise, should be recommended to decrease the risk of developing abnormalities of glucose and BP.

## Key Points

**Question:**

Is the metabolically healthy overweight phenotype associated with metabolic abnormalities?

**Findings:**

In this cohort study of 3204 Chinese adults, metabolically healthy overweight was associated with high future risk of glucose abnormality and high blood pressure.

**Meaning:**

These findings suggest that more attention should be given to this unique subtype of overweight and obesity if the results are replicated in additional studies.

## Teaser

This cohort study assesses the association of metabolically healthy overweight phenotype with the risk of glucose level abnormality and high blood pressure in Chinese adults.

## Introduction

The high prevalence of obesity has become an increasing costly burden on both the Asian and the Western health care systems during the past century.^1^ Obesity is associated with a wide range of metabolic complications, including type 2 diabetes, hypertension, stroke, depression, and certain types of cancer, thus causing a burden to public health.^2,3^

However, some studies^4,5^ have found that obesity does not always entail metabolic abnormalities and does not necessarily increase the risk of cardiometabolic complications and mortality. The term *metabolically healthy overweight* (MHO) has been proposed to describe the unique phenotype of overweight and obese individuals with normal glucose level, blood pressure (BP), and lipid level.^6^ However, some knowledge gaps need to be addressed. Some^7,8,9,10^ but not all studies^11,12,13,14^ found a significant association between MHO and higher risk of metabolic complications. Thus, conclusive results could not be generated as to whether the MHO phenotype is a transitional stage or resistant to metabolic abnormalities based on current evidence. Furthermore, the definition of MHO differs greatly among studies.^15^ Even in the same population, different criteria of MHO generate mixed results.^16^ In addition, some metabolic abnormalities, such as hyperuricemia^17^ and fatty liver,^18^ were not taken into consideration in any of the aforementioned studies.^7,8,9,10,11,12,13,14,15,16,17,18^ Therefore, we aimed to evaluate the association between the MHO phenotype and the risk of incident glucose level abnormality and high BP in Chinese adults during 4 years of follow-up.

## Methods

### Study Population

This cohort study included participants recruited from the Health Management Center at Ren Ji Hospital, Shanghai, China, from January 1, 2013, to October 31, 2018. All adults (18-100 years of age) receiving a routine health checkup at the Health Management Center from January 1 to December 31, 2013, were eligible for the study. The study protocol was approved by the Ethical Committee of Ren Ji Hospital, School of Medicine, Shanghai Jiao Tong University. As a deidentified secondary data analysis, patient consent was waived by the Ethical Committee. This study followed the Strengthening the Reporting of Observational Studies in Epidemiology (STROBE) reporting guideline.

The initial recruitment resulted in the identification of 55 155 individuals. Body weight, the concentrations of hemoglobin A_1c_ (HbA_1c_) and fasting blood glucose (FBG), and systolic and diastolic BP were annually measured during the subsequent 5 years. To recruit only participants who were metabolically healthy, we excluded 9212 participants with a history of chronic metabolic diseases and cancer. Then we excluded 31 415 participants with metabolic abnormalities (high BP, impaired glucose regulation, elevated concentration of triglycerides, and decreased concentration of high-density lipoprotein cholesterol) based on a joint statement.^19^ A total of 2414 individuals with total cholesterol level abnormalities, 270 with low-density lipoprotein level abnormalities, 270 with hyperuricemia, and 1212 with fatty liver abnormalities were also considered metabolically unhealthy^18,20^ and were excluded. Finally, we excluded 7158 participants who were unavailable for follow-up. The final sample size was 3204 metabolically healthy Chinese adults (Figure). Participants included in the study tended to be younger, to more likely be women, and to have lower concentrations of HbA_1c_ and FBG and lower BP at baseline compared with those who were excluded (eTable 1 in the Supplement).

**Figure.**
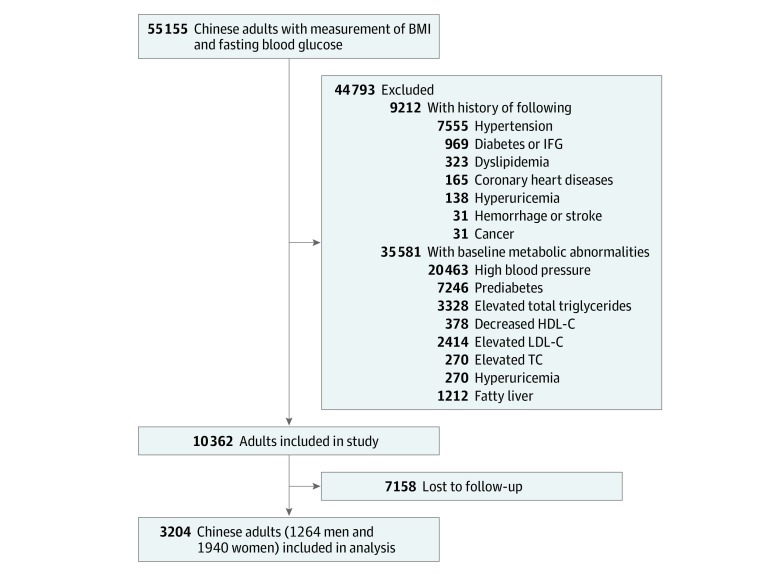
Sample Recruitment Process BMI indicates body mass index; HDL-C, high-density lipoprotein cholesterol; IFG, impaired fasting glucose; LDL-C, low-density lipoprotein cholesterol; and TC, triglycerides.

### Assessment of FBG Levels, HbA_1c_ Levels, and Blood Pressure

Venous blood samples were obtained and transfused into vacuum tubes containing EDTA in the morning after participants fasted for 6 hours. The whole blood samples were stored at 4 °C for further analysis. An automatic analyzer (Roche 701 Bioanalyzer) was used to measure FBG with the hexokinase/glucose-6-phosphate dehydrogenase method. The coefficient of variation using blind quality control specimens was 2.0%. The concentration of HbA_1c_ was measured by high-performance liquid chromatography using the fully automated VARIANT II Hemoglobin Testing System (Bio-Rad). The measurement range was 2.0% to 18.0%. Glucose abnormality was confirmed if the FBG level was 101 mg/dL or higher (to convert to millimoles per liter, multiply by 0.0555) or the HbA_1c_ level was 5.7% or higher (to convert to proportion of hemoglobin, multiply by 0.01) at least twice during the subsequent 4 years of follow-up.^21^

Blood pressure was measured twice using an automatic BP meter (HBP-9020, Omron Co Ltd) after participants were seated for at least 10 minutes. The mean of 2 measurements was recorded for further analysis. High BP was confirmed if systolic BP was 130 mm Hg or higher or diastolic BP was 80 mm Hg or higher at least twice during the subsequent 4 years of follow-up.^22^

### Assessment of Body Weight and Height

Body weight (to the nearest 0.5 kg) and height (to the nearest 0.5 cm) were measured with the patient in the standing position without shoes and in light clothing by using an electronic scale (SK-CK; Shuang Jia Company). Body mass index (BMI) was calculated as weight in kilograms divided by height in meters squared. Metabolically healthy overweight was defined as a BMI of 24.0 in 2013 (baseline) and 2014,^23^ and the remaining participants were considered to be metabolically healthy normal weight (MHN).

### Assessment of Other Confounders

The level of high-sensitivity C-reactive protein was measured by the immunoturbidimetric method, whereas serum insulin level was measured by the immunoassay method. Total cholesterol, triglycerides, high-density lipoprotein cholesterol, low-density lipoprotein cholesterol, alanine aminotransferase, aspartate aminotransferase, creatinine, and uric acid levels were measured using an automatic biochemical analyzer (Roche 701 Bioanalyzer; Roche). All the measurements were completed in the Clinical Laboratory of Ren Ji Hospital. The estimated glomerular filtration rate was calculated using the Chronic Kidney Disease Epidemiology Collaboration 2-level race equation.^24^ The homeostasis model assessment (HOMA) index was calculated using the following equation: HOMA index = [fasting serum insulin × fasting glucose]/22.5. Participants were confirmed as having insulin resistance if their HOMA index was in the top quartile of the distribution among nondiabetic individuals.^25^ Data on the history of hypertension, diabetes or impaired FBG, dyslipidemia, hyperuricemia, stroke and hemorrhage, and coronary heart diseases (coronary atherosclerosis, coronary artery bypass grafting, stent surgery, and ischemic infarction) were collected using a self-report questionnaire.

### Statistical Analysis

We completed all statistical analyses using SAS statistical software, version 9.4 (SAS Institute Inc). Formal hypothesis testing was 2-sided with a significance level of *P* < .05. Because abnormalities of glucose and BP were confirmed at least twice during the 4 years of follow-up, the person-time of follow-up for each participant was determined from January 1, 2014, to the first onset date of the outcomes (incident glucose abnormality and high BP) or the end of follow-up (December 31, 2018), whichever came first.

We used the Cox proportional hazards regression model to evaluate the association between MHO phenotype and the assessed outcomes. We adjusted the potential confounders in 2 different models: model 1 adjusted for age and sex and model 2 further adjusted for systolic BP; diastolic BP; FBG, HbA_1c_, total cholesterol, triglycerides, low-density lipoprotein cholesterol, high-density lipoprotein cholesterol, alanine aminotransferase, aspartate aminotransferase, and uric acid levels; and estimated glomerular filtration rate. We further adjusted for baseline high-sensitivity C-reactive protein and HOMA index to assess whether the potential association between MHO phenotype and the outcomes was related to inflammatory status and insulin resistance.

Likelihood ratio tests were conducted to examine statistical interactions among MHO, sex, and age (<65 vs ≥65 years) in association with abnormalities of glucose and BP by comparing −2 log likelihood χ^2^ between nested models with and without the cross-product terms. To test the robustness of the main results, we conducted 5 sensitivity analyses in model 2. We used the cumulative mean BMI during follow-up as the exposure. Participants were also classified into the 2 following groups based on their cumulative mean BMI: MHN (BMI, 18.5-23.9) and MHO (BMI, ≥24.0).^23^ Then we censored older participants (≥65 years of age), with overweight in 2013 or 2014, elevated high-sensitivity C-reactive protein level (≥1.0 mg/L [to convert to nanomoles per liter, multiply by 9.524]),^26^ and insulin resistance (HOMA index, ≥75th percentile).^25^

## Results

A total of 3204 metabolically healthy Chinese adults (mean [SD] age, 39.8 [10.9] years; 1940 women [60.5%]) were included in the study. The prevalence of MHO was 7.0%. The mean (SD) participant findings were as follows: BMI, 21.8 (2.5); FBG level, 86 (7) mg/dL; HbA_1c_ level, 5.1% (0.2%); systolic BP, 109.5 (9.6) mm Hg; and diastolic BP, 67.2 (7.0) mm Hg. Metabolically healthy overweight was associated with mean (SD) baseline age (42.5 [10.6] years for MHO vs 39.6 [10.9] years for MHN; *P* < .001), systolic BP (113.0 [8.9] mm Hg for MHO vs 109.2 [9.6] mm Hg for MHN; *P* < .001), diastolic BP (69.3 [6.7] mm Hg for MHO vs 67.0 [7.0] mm Hg for MHN; *P* < .001), HOMA index (1.3 [0.6] for MHO vs 1.0 [0.6] for MHN; *P* < .001), alanine aminotransferase (19.2 [12.0] U/L for MHO vs 16.3 [13.6] U/L for MHN; *P* = .002 [to convert aminotransferase to microkatals per liter, multiply by 0.0167]), high-density lipoprotein cholesterol (54 [12] mg/dL for MHO vs 62 [12] for MHN; *P* < .001) and low-density lipoprotein cholesterol (100 [19] mg/dL for MHO vs 97 [19] mg/dL for MHN; *P* = .003 [to convert high-density lipoprotein and low-density lipoprotein cholesterol to millimoles per liter, multiply by 0.0259]), and uric acid levels (5.2 [1.2] mg/dL for MHO vs 4.8 [1.2] mg/dL for MHN; *P* < .001 [to convert uric acid to micromoles per liter, multiply by 59.485]) (Table 1).

**Table 1.** Baseline Characteristics Across Different Baseline Body Weight Status in 3204 Chinese Adults^a^

Characteristic	MHN Group (n = 2981)	MHO Group (n = 223)	*P* Value
Sex, No. (%)			
Men	1133 (38.0)	131 (58.7)	<.001
Women	1848 (62.0)	92 (41.3)	
Age, y	39.6 (10.9)	42.5 (10.6)	<.001
Blood pressure, mm Hg			
Systolic	109.2 (9.6)	113.0 (8.9)	<.001
Diastolic	67.0 (7.0)	69.3 (6.7)	<.001
Fasting blood glucose level, mg/dL	86 (7)	86 (5)	.34
Hemoglobin A_1c_ level, %	5.1 (0.2)	5.2 (0.3)	.16
HOMA index^b^	1.0 (0.6)	1.3 (0.6)	<.001
Aminotransferase level, U/L			
Alanine	16.3 (13.6)	19.2 (12.0)	.002
Aspartate	18.2 (8.3)	18.2 (5.7)	.91
Cholesterol level, mg/dL			
Total	174 (23)	174 (23)	.89
Triglycerides	80 (27)	88 (27)	<.001
High-density lipoprotein	62 (12)	54 (12)	<.001
Low-density lipoprotein	97 (19)	100 (19)	.003
Uric acid level, mg/dL	4.8 (1.2)	5.2 (1.2)	<.001
Estimated glomerular filtration rate, mL/min/1.73 m^2^	123.9 (26.1)	120.7 (25.9)	.62

Abbreviations: HOMA, homeostasis model assessment; MHN, metabolically healthy normal weight; MHO, metabolically healthy overweight.

SI conversion factors: to convert fasting blood glucose to millimoles per liter, multiply by 0.0555; hemoglobin A_1c_ to proportion of hemoglobin, multiply by 0.01; aminotransferase to microkatals per liter, multiply by 0.0167; total, high-density lipoprotein, and low-density lipoprotein cholesterol to millimoles per liter, multiply by 0.0259; triglycerides to millimoles per liter, multiply by 0.0113; and uric acid to micromoles per liter, multiply by 59.485.

^a^ Data are presented as mean (SD) unless otherwise indicated. Metabolically healthy overweight was defined as a body mass index (calculated as weight in kilograms divided by height in meters squared) of 24.0 in 2013 (baseline) and 2014, and the remaining participants were considered metabolically healthy normal weight.

^b^ The HOMA index was calculated as follows: HOMA index = [fasting serum insulin × fasting glucose]/22.5.

We identified 146 incident cases of glucose level abnormality and 220 cases of high BP during 4 years of follow-up. Compared with MHN, MHO was associated with a high risk of glucose level abnormality (adjusted hazard ratio [HR], 2.36; 95% CI, 1.52-3.64) and high BP (adjusted HR, 1.73; 95% CI, 1.18-2.53) after adjusting for several potential confounders (Table 2). Further adjusting for baseline high-sensitivity C-reactive protein level (for glucose abnormality: adjusted HR, 2.36; 95% CI, 1.52-3.68; for high BP: adjusted HR, 1.65; 95% CI, 1.12,-2.44) and HOMA insulin resistance index (for glucose abnormality: adjusted HR, 2.58; 95% CI, 1.65-4.04; for high BP: adjusted HR, 1.83; 95% CI, 1.23-2.71), the association between MHO and abnormalities of glucose and BP did not change (Table 2).

**Table 2.** Risks of Incident Glucose Abnormality and High Blood Pressure by Body Weight Status During 4-Year Follow-up Among 3204 Chinese Adults^a^

Outcome	Adjusted HR (95% CI)	
	MHN Group (n = 2981)	MHO Group (n = 223)
**Glucose Level Abnormality**		
Participants, No. (%)	2981 (93.0)	223 (7.0)
Incident cases, No. (%)	119 (81.5)	27 (18.5)
Age- and sex-adjusted model	1 [Reference]	2.47 (1.62-3.77)
Multiple-adjusted model^b^	1 [Reference]	2.36 (1.52-3.64)
Further adjusting for baseline hs-CRP	1 [Reference]	2.36 (1.52-3.68)
Further adjusting for baseline HOMA index^c^	1 [Reference]	2.58 (1.65-4.04)
**High Blood Pressure**		
Participants, No. (%)	2981 (93.0)	223 (7.0)
Incident cases, No. (%)	185 (84.1)	35 (15.9)
Age- and sex-adjusted model	1 [Reference]	2.18 (1.52-3.14)
Multiple-adjusted model^b^	1 [Reference]	1.73 (1.18-2.53)
Further adjusting for baseline hs-CRP	1 [Reference]	1.65 (1.12-2.44)
Further adjusting for baseline HOMA index^c^	1 [Reference]	1.83 (1.23-2.71)

Abbreviations: HOMA, homeostasis model assessment; HR, hazard ratio; hs-CRP, high-sensitivity C-reactive protein; MHN, metabolically healthy normal weight; MHO, metabolically healthy overweight.

^a^ Metabolically healthy overweight was defined as a body mass index (calculated as weight in kilograms divided by height in meters squared) of 24.0 in 2013 (baseline) and 2014, and the remaining participants were considered metabolically healthy normal weight.

^b^ Multiple-adjusted model was adjusted for the following: age; sex; systolic blood pressure; diastolic blood pressure; fasting blood glucose, hemoglobin A_1c_, total cholesterol, triglycerides, low-density lipoprotein cholesterol, high-density lipoprotein cholesterol, alanine aminotransferase, aspartate aminotransferase, and uric acid levels; and estimated glomerular filtration rate.

^c^ The HOMA index was calculated as follows: HOMA index = [fasting serum insulin × fasting glucose]/22.5.

Sex and age modified the association between MHO and abnormality of glucose levels, whereas sex but not age modified the association between MHO and high BP (eTable 2 in the Supplement). Use of the cumulative mean BMI as the exposure (for glucose abnormality: adjusted HR, 2.33; 95% CI, 1.60-3.38; for high BP: adjusted HR, 2.50; 95% CI, 1.83-3.41) or excluding older participants (for glucose abnormality: adjusted HR, 2.43; 95% CI, 1.54-3.85; for high BP: adjusted HR, 1.88; 95% CI, 1.27-2.77), with elevated high-sensitivity C-reactive protein levels (for glucose abnormality: adjusted HR, 2.14; 95% CI, 1.21-3.78; for high BP: adjusted HR, 1.85; 95% CI, 1.18-2.92), insulin resistance (for glucose abnormality: adjusted HR, 2.11; 95% CI, 1.21-3.67), and overweight in 2013 (baseline) or 2014 (for glucose abnormality: adjusted HR, 2.20; 95% CI, 1.41-3.42; for high BP: adjusted HR, 1.60; 95% CI, 1.09-2.36) generated similar results except for an association between MHO and high BP (HR, adjusted 1.61; 95% CI, 0.99-2.62) after excluding those with insulin resistance (Table 3 and Table 4).

**Table 3.** Sensitivity Analyses of Risks of Incident Glucose Abnormality by Body Weight Status During 4-Year Follow-up Among 3204 Chinese Adults^a^

Sensitivity Analysis	MHN Group (n = 2981)	MHO Group (n = 223)
**Sensitivity Analysis 1**^b^		
Participants, No. (%)	2797 (87.3)	407 (12.7)
Cases of glucose level abnormality, No. (%)	97 (66.4)	49 (33.6)
Multiple-adjusted model adjusted hazard ratio (95% CI)^c^	1 [Reference]	2.33 (1.60-3.38)
**Sensitivity Analysis 2**^d^		
Participants, No. (%)	2855 (92.9)	217 (7.1)
Cases of glucose level abnormality, No. (%)	106 (80.9)	25 (19.1)
Multiple-adjusted model adjusted hazard ratio (95% CI)^c^	1 [Reference]	2.43 (1.54-3.85)
**Sensitivity Analysis 3**^e^		
Participants, No. (%)	2505 (94.2)	153 (5.8)
Cases of glucose level abnormality, No. (%)	93 (86.1)	15 (13.9)
Multiple-adjusted model adjusted hazard ratio (95% CI)^c^	1 [Reference]	2.14 (1.21-3.78)
**Sensitivity Analysis 4**^f^		
Participants, No. (%)	2295 (94.5)	133 (5.5)
Cases of glucose level abnormality, No. (%)	94 (85.5)	16 (14.5)
Multiple-adjusted model adjusted hazard ratio (95% CI)^c^	1 [Reference]	2.11 (1.21-3.67)
**Sensitivity Analysis 5**^g^		
Participants, No. (%)	2711 (92.4)	223 (7.6)
Cases of glucose level abnormality, No. (%)	110 (80.3)	27 (19.7)
Multiple-adjusted model adjusted hazard ratio (95% CI)^c^	1 [Reference]	2.20 (1.41-3.42)

Abbreviations: MHN, metabolically healthy normal weight; MHO, metabolically healthy overweight.

^a^ Metabolically healthy overweight was defined as a body mass index (calculated as weight in kilograms divided by height in meters squared) of 24.0 in 2013 (baseline) and 2014, and the remaining participants were considered metabolically healthy normal weight.

^b^ Sensitivity analysis 1 used the cumulative mean body mass index (2013-2018) as the exposure.

^c^ Multiple-adjusted model was adjusted for the following: age; sex; systolic blood pressure; diastolic blood pressure; fasting blood glucose, hemoglobin A_1c_, total cholesterol, triglycerides, low-density lipoprotein cholesterol, high-density lipoprotein cholesterol, alanine aminotransferase, aspartate aminotransferase, and uric acid levels; and estimated glomerular filtration rate.

^d^ Sensitivity analysis 2 excluded older adults (n = 132).

^e^ Sensitivity analysis 3 excluded participants with high C-reactive protein levels (≥1 mg/L) at baseline (n = 546).

^f^ Sensitivity analysis 4 excluded participants with insulin resistance (homeostasis assessment model index ≥75th percentile) at baseline (n = 776).

^g^ Sensitivity analysis 5 excluded participants with overweight in 2013 (baseline) or 2014 (n = 270).

**Table 4.** Sensitivity Analyses of Risks of Incident High Blood Pressure by Body Weight Status During 4-Year Follow-up Among 3204 Chinese Adults^a^

Sensitivity Analysis	MHN Group (n = 2981)	MHO Group (n = 223)
**Sensitivity Analysis 1**^b^		
Participants, No. (%)	2797 (87.3)	407 (12.7)
Cases of high blood pressure, No. (%)	147 (66.8)	73 (33.2)
Multiple-adjusted model adjusted hazard ratio (95% CI)^c^	1 [Reference]	2.50 (1.83-3.41)
**Sensitivity Analysis 2**^d^		
Participants, No. (%)	2855 (92.9)	217 (7.1)
Cases of high blood pressure, No. (%)	163 (82.7)	34 (17.3)
Multiple-adjusted model adjusted hazard ratio (95% CI)^c^	1 [Reference]	1.88 (1.27-2.77)
**Sensitivity Analysis 3**^e^		
Participants, No. (%)	2505 (94.2)	153 (5.8)
Cases of high blood pressure, No. (%)	154 (86.5)	24 (13.5)
Multiple-adjusted model adjusted hazard ratio (95% CI)^c^	1 [Reference]	1.85 (1.18-2.92)
**Sensitivity Analysis 4**^f^		
Participants, No. (%)	2295 (94.5)	133 (5.5)
Cases of high blood pressure, No. (%)	141 (87. 0)	21 (13.0)
Multiple-adjusted model adjusted hazard ratio (95% CI)^c^	1 [Reference]	1.61 (0.99-2.62)
**Sensitivity Analysis 5**^g^		
Participants, No. (%)	2711 (92.4)	223 (7.6)
Cases of high blood pressure, No. (%)	167 (80.3)	35 (19.7)
Multiple-adjusted model adjusted hazard ratio (95% CI)^c^	1 [Reference]	1.60 (1.09-2.36)

Abbreviations: MHN, metabolically healthy normal weight; MHO, metabolically healthy overweight.

^a^ Metabolically healthy overweight was defined as a body mass index (calculated as weight in kilograms divided by height in meters squared) of 24.0 in 2013 (baseline) and 2014, and the remaining participants were considered metabolically healthy normal weight.

^b^ Sensitivity analysis 1 used the cumulative mean body mass index (2013-2018) as the exposure.

^c^ Multiple-adjusted model was adjusted for the following: age; sex; systolic blood pressure; diastolic blood pressure; fasting blood glucose, hemoglobin A_1c_, total cholesterol, triglycerides, low-density lipoprotein cholesterol, high-density lipoprotein cholesterol, alanine aminotransferase, aspartate aminotransferase, and uric acid levels; and estimated glomerular filtration rate.

^d^ Sensitivity analysis 2 excluded older adults (n = 132).

^e^ Sensitivity analysis 3 excluded participants with high C-reactive protein levels (≥1 mg/L) at baseline (n = 546).

^f^ Sensitivity analysis 4 excluded participants with insulin resistance (homeostasis assessment model index ≥75th percentile) at baseline (n = 776).

^g^ Sensitivity analysis 5 excluded participants with overweight in 2013 (baseline) and 2014 (n = 270).

## Discussion

In the current prospective cohort study, we found that MHO was associated with glucose level abnormality and high BP in 3204 Chinese adults without a history of major metabolic diseases and whose blood glucose level, HbA_1c_ level, BP, lipid profile, uric acid level, and liver ultrasonographic findings were normal at baseline. The key strength of our research was the strict exclusion of people with a range of metabolic disorders, which provided greater breadth of insights regarding accurate estimates of metabolic outcomes of the MHO phenotype.

The lack of a consensus definition of *metabolically healthy* was often given as a reason for the discrepancies of the associations between MHO and metabolic abnormalities among previous studies. Even in the same study population, different criteria of MHO could generate mixed results.^16^ Different criteria have been used depending on the number of abnormalities (eg, 0-1, ≤2, and ≤3), HOMA index, and the combination.^15,27^ Usually, metabolic abnormalities have been defined based on the FBG level, BP, and lipid profile. However, determinants of MHO should not be limited to these factors only. For example, hyperuricemia^17^ and fatty liver^18^ have also been associated with metabolic abnormalities. Furthermore, it is doubtful whether an individual with 1 type of metabolic abnormality, such as high BP, could be considered as metabolically healthy.

To eliminate the potential bias associated with the disagreement of the definition, we defined metabolically healthy as follows: no history of diabetes or impaired FBG, hypertension, dyslipidemia, cardiovascular heart diseases, stroke or hemorrhage, hyperuricemia, or cancer and no metabolic abnormalities based on FBG level, HbA_1c_ level, BP, lipid profile, serum uric acid level, and liver ultrasonographic findings at baseline. To lower the possibility of misclassification, we used repeated measurements (at baseline and in 2014) to define MHO. To our knowledge, the criterion might be the strictest one compared with those in the previous studies.^6,7,15^ We found that MHO was associated with glucose level abnormality and high BP after adjusting for conventional risk factors and further adjusting for HOMA index and high-sensitivity C-reactive protein level. Our results are supported by another large cohort study^10^ (3.5 million adults without a history of cardiovascular vascular disease), which also defined metabolically healthy as having no metabolic abnormalities. The authors of that study^10^ reported that the MHO phenotype was associated with cardiovascular disease events during a mean follow-up of 5.4 years compared with the normal-weight phenotype. Our results, together with those of the aforementioned cohort study^10^ and a previous review,^28^ suggest that overweight individuals who are metabolically healthy may have a high risk of developing metabolic abnormalities in the future.

Because BMI is a protective factor against mortality in the elderly population,^5^ age can be a significant confounder in developing BMI-associated metabolic abnormalities. Thus, to avoid the age interaction, a sensitivity analysis was conducted by stratifying MHO outcomes by age. The results indicated that the association between MHO and glucose abnormality was only significant for those younger than 65 years. The mechanism underlying the complexity of BMI-related risk remained unclear; however, the reason might be associated with BMI having limited value in distinguishing between lean and fat mass.^29^

### Limitations

This study has several limitations. First, the use of medication was scarce. However, we excluded participants with a self-reported history of a series of metabolic diseases, which might mitigate the association with medication. Second, behavior habit, such as diet and physical activities, were not collected in the current analysis. We thus could not analyze the extent to which adjustment for diet and physical activity would have modified the association between MHO and the outcomes. Moreover, the duration of follow-up was relatively short. Also, waist circumference measurements were not collected. A single use of BMI might lead to controversial results.^30^ Prospective studies with a representative population and deliberate collection of information about potential confounders are warranted to confirm the association of MHO with health.

## Conclusions

Metabolically healthy overweight was associated with a high future risk of glucose level abnormality and high BP in Chinese adults. These findings suggest that more attention should be given to this unique subtype of overweight and obesity if the results are replicated in additional studies.

## Notes

Supplement. **eTable 1.** Baseline Characteristics of Included and Excluded Study Participants **eTable 2.** Adjusted Hazard Ratios and 95% Confidence Intervals for Risks of Incident Abnormalities of Glucose and Blood Pressure Across Different Body Weight Status During Four-Year Follow-up Among 3204 Chinese Adults: Subgroup Analysis
