# Supplementary material for: Association of Metabolically Healthy Overweight Phenotype With Abnormalities of Glucose Levels and Blood Pressure Among Chinese Adults
Source: JAMA Netw Open. 2019 Oct 25;2(10):e1914025. doi: 10.1001/jamanetworkopen.2019.14025 (PMC6822082; doi:10.1001/jamanetworkopen.2019.14025)
Supplement: Supplement. — eTable 1. Baseline Characteristics of Included and Excluded Study Participants eTable 2. Adjusted Hazard Ratios and 95% Confidence Intervals for Risks of Incident Abnormalities of Glucose and Blood Pressure Across Different Body Weight Status During Four-Year Follow-up Among 3204 Chinese Adults: Subgroup Analysis [file jamanetwopen-2-e1914025-s001.pdf]

Supplementary Online Content

Xu R, Gao X, Wan Y, Fan Z. Association of metabolically healthy overweight phenotype with abnormalities of glucose and blood pressure among Chinese adults. *JAMA Netw Open*. 2019;2(10):e1914025. doi:10.1001/jamanetworkopen.2019.14025

eTable 1. Baseline Characteristics of Included and Excluded Study Participants

eTable 2. Adjusted Hazard Ratios and 95% Confidence Intervals for Risks of Incident Abnormalities of Glucose and Blood Pressure Across Different Body Weight Status During Four-Year Follow-up Among 3204 Chinese Adults: Subgroup Analysis

This supplementary material has been provided by the authors to give readers additional information about their work.

© 2019 Xu R et al. *JAMA Network Open*.

eTable 1. Baseline Characteristics of Included and Excluded Study Participants

Variables	Participants in the study	Participants out of the study	P value
Sample	3,204	51,951	--
Age, y	42.4±11.6	50.7±14.8	<0.001
Sex, men/women, n (%)	1264 (39.4)/1940 (54.8)	31,067 (59.8)/20,884 (40.2)	<0.001
hs-CRP, mg/L	1.1±3.6	1.6±4.1	<0.001
BMI, kg/m^2	22.3±2.8	24.3±3.3	<0.001
SBP, mmHg	113.3±13.0	128.3±19.1	<0.001
DBP, mmHg	68.9±8.6	77.1±12.4	<0.001
FBG, mmol/L	5.0±0.7	5.4±1.3	<0.001
HbA1c, %	5.2±0.4	5.6±0.8	<0.001
HOMA-IR	1.2±0.8	1.8±1.6	<0.001
TC, mmol/L	4.7±0.7	5.1±1.0	<0.001
TG, mmol/L	1.0±0.7	1.7±1.4	<0.001
HDL-C, mmol/L	1.5±0.3	1.4±0.4	<0.001
LDL-C, mmol/L	2.6±0.6	3.0±0.8	<0.001
Uric acid, μmol/L	295.5±73.1	338.7±86.1	<0.001
eGFR, ml/min/1.73m^2	125.7±82.7	109.7±24.7	<0.001

Abbreviation: **hs-CRP**, high sensitivity C-reactive protein; **HbA1c**, glycated hemoglobin A1c; **BMI**, body mass index; **SBP**, systolic blood pressure; **DBP**, diastolic blood pressure; **FBG**, fasting blood glucose; **TC**, total cholesterol; **TG**, triglyceride; **HDL-C**, high density lipoprotein cholesterol; **LDL-C**, low density lipoprotein cholesterol; **eGFR**, estimating glomerular filtration rate.

© 2019 Xu R et al. *JAMA Network Open*.

eTable 2. Adjusted Hazard Ratios and 95% Confidence Intervals for Risks of Incident Abnormalities of Glucose and Blood Pressure Across Different Body Weight Status During Four-Year Follow-up Among 3204 Chinese Adults: Subgroup Analysis

Outcome	Group variables	Model	MHN	MHO
Glucose abnormality	Men	Number of participants	1,133 (89.6%)	131 (10.4%)
		Number of incident cases	77 (81.0%)	18 (19.0%)
		Multiple-adjusted model	**Ref (1.0)**	2.06 (1.21, 3.52)
	Women	Number of participants	1,848 (95.3%)	92 (4.7%)
		Number of incident cases	42 (82.4%)	9 (17.6%)
		Multiple-adjusted model	**Ref (1.0)**	3.63 (1.72, 7.65)
	<65 years	Number of participants	2,855 (92.9%)	217 (7.1%)
		Number of incident cases	106 (80.9%)	25 (19.1%)
		Multiple-adjusted model	**Ref (1.0)**	2.55 (1.62, 7.65)
	≥65 years	Number of participants	126 (95.5%)	6 (4.5%)
		Number of incident cases	13 (86.7%)	2 (13.3%)
		Multiple-adjusted model	**Ref (1.0)**	5.55 (0.7, 42.88)
High blood pressure	Men	Number of participants	1,133 (89.6%)	131 (10.4%)
		Number of incident cases	114 (83.8%)	22 (16.2%)
		Multiple-adjusted model	**Ref (1.0)**	1.39 (0.86, 2.26)
	Women	Number of participants	1,848 (95.3%)	92 (4.7%)
		Number of incident cases	71 (84.5%)	13 (15.5%)
		Multiple-adjusted model	**Ref (1.0)**	2.66 (1.43, 4.95)
	<65 years	Number of participants	2,855 (92.9%)	217 (7.1%)
		Number of incident cases	163 (82.7%)	34 (17.3%)
		Multiple-adjusted model	**Ref (1.0)**	2.05 (1.39, 3.01)
	≥65 years	Number of participants	126 (95.5%)	6 (4.5%)
		Number of incident cases	22 (95.7%)	1 (4.3%)
		Multiple-adjusted model	**Ref (1.0)**	1.75 (0.18, 17.27)

Note:

1. "MHO was defined if participants were confirmed with overweight (BMI≥24.0 kg/m^2) both in 2013 (baseline) and in 2014, and the

© 2019 Xu R et al. *JAMA Network Open*.

remained participants were defined as MHN.

2. Multiple-adjusted model: adjusting for age (y), systolic blood pressure (mmHg), diastolic blood pressure (mmHg), fasting blood glucose (mmol/L), glycated hemoglobin A1c (%), total cholesterol (mmol/L), triglyceride (mmol/L), low-density-lipoprotein cholesterol (mmol/L), high-density-lipoprotein cholesterol (mmol/L), alanine transferase (IU/l), aspartate transferase (IU/l), uric acid (μmol/L), and eGFR(ml/min/1.73m^2). However, group variables were not included in the model.

© 2019 Xu R et al. *JAMA Network Open*.
